# Ecological and Ethological Assessment of Captive *Testudo graeca* in an Urban Bazaar: A Case of High-Constraint Wildlife Tourism in Kastamonu, Northern Anatolia

**DOI:** 10.3390/ani16081141

**Published:** 2026-04-09

**Authors:** Murat Afsar, Çetin Çelik, Mahsun Cağlar, Pınar Durmuş, Birgül Afsar

**Affiliations:** 1 Department of Biology, Zoology Section, Faculty of Engineering and Natural Sciences, Manisa Celal Bayar University, Yunusemre 45140, Türkiye; birgul.afsar@cbu.edu.tr; 2 Graduate School of Natural and Applied Sciences, Manisa Celal Bayar University, Yunusemre 45140, Türkiyemahsun_caglarr@hotmail.com (M.C.);; 3 Akhisar Science and Art Center, Akhisar 45200, Türkiye

**Keywords:** *Testudo graeca*, wildlife tourism ethics, ethogram, carrying capacity, commodification, ethnozoology, Kastamonu

## Abstract

The Spur-thighed tortoise (*Testudo graeca*) is a long-lived terrestrial reptile listed as ‘Vulnerable’ on the IUCN Red List and protected under CITES Appendix II. As an ecosystem engineer, it plays a vital role in Mediterranean landscapes, yet it frequently faces anthropogenic pressures in urban environments. This study provides an ecological and ethological assessment of a captive *T. graeca* population (n = 42) in the historical Münire Madrasa Handicrafts Bazaar in Kastamonu, Türkiye. The methodology integrated spatial carrying capacity modeling (Boullon model), systematic ethogram-based observations (120 h), and ethnozoological surveys (n = 200). Spatial analysis revealed that the population exceeds the corrected Real Carrying Capacity (RCC ≈ 10) by four times (Overcapacity Index: 4.2) within the 70 m^2^ area. Ethological findings documented chronic stress, with stereotypic pacing (H1) occupying 32% of the time budget, alongside a significant loss of anti-predator mechanisms due to anthropogenic habituation (İ1). While stakeholders (100%, 95% CI: 98.1–100%) perceive the tortoises as cultural symbols of abundance, the biological reality indicates severe welfare risks, including potential metabolic bone disease from a monotonous anthropogenic diet and a disrupted Ca:P ratio. The site is categorized as a ‘High-Constraint Interaction Zone’. We propose a management transition toward a monitored ‘Urban Wildlife Education Station’ to align local cultural values with international animal welfare and conservation standards.

## Simple Summary

This study investigates the lives of 42 tortoises (*Testudo graeca*) kept in a historical bazaar in Kastamonu, Türkiye. While local residents and visitors perceive the tortoises as symbols of abundance and believe they are well-cared for, our scientific findings tell a different story. By measuring the physical space and the animals’ behavior, we discovered that the area is severely overcrowded—hosting four times more tortoises than the environment can naturally support. This overcrowding and constant human contact cause the tortoises chronic stress, leading to repetitive behaviors and health risks from an unsuitable diet of lettuce and cucumbers. We conclude that the current situation harms the animals’ welfare. We recommend transforming this site into a professional education station where the tortoises can receive proper nutrition and care under expert supervision, balancing cultural traditions with modern animal welfare standards.

## 1. Introduction

Anatolia is a geography where human–animal interaction possesses a millennial depth. Recent ethnozoological studies in Eastern Anatolia demonstrate that tortoises are not merely ecological figures but also medicinal and magical entities. As documented by [1], traditional vertebrate-oriented practices show that local populations regard these creatures as elements of “healing” and “protection.” Similarly, research in Southeastern Anatolia indicates that tortoises hold a place in the cosmology of nomadic communities as symbols of “unshakeability” and “eternity” [2]. In contrast, these complex cultural understandings often operate independently of, and at times in direct contradiction to, the species’ fundamental biological requirements. Understanding these cultural perceptions, therefore, requires a firm grounding in the species’ actual biological reality.

However, understanding these cultural perceptions requires a grounding in the species’ actual biological reality. The Spur-thighed tortoise (*Testudo graeca*) is a wide-ranging polytypic species found across North Africa, Southern Europe, and Western Asia, with Türkiye serving as a major stronghold where it is distributed across almost all regions [3,4]. Within its Anatolian range, the species occupies diverse habitats from sea level up to 2700 m, ranging from coastal dunes and scrublands to dry open steppes [5]. Habitat preferences are often linked to sclerophyllous vegetation, where individuals utilize seasonal micro-habitats such as *Sarcopoterium* or *Juncus* for thermoregulation [6]. The Spur-thighed tortoise (*Testudo graeca*) is a long-lived terrestrial reptile listed as ‘Vulnerable’ on the IUCN Red List and protected under CITES Appendix II. As an ecosystem engineer, *T. graeca* plays a vital role in seed dispersal and soil health within Mediterranean and steppic landscapes [7]. Its high sensitivity to habitat fragmentation and anthropogenic disturbance makes it a key indicator species for assessing the ecological integrity of protected and urban-fringe areas. However, its longevity and docile nature often make it a primary target for illegal trade and ‘well-intentioned’ but biologically unsuitable captive displays in urban settings.

Physiologically, *T. graeca* exhibits specific thermal limits; individuals initiate respiratory cooling above 30 °C and reach critical panting thresholds beyond 35 °C [8]. In their natural environment, these tortoises require extensive home ranges—averaging 1.48 to 10 hectares—to satisfy their biological and reproductive needs [9,10]. Despite this adaptability, the species is currently classified as Vulnerable (VU) on the IUCN Red List [7]. This status is justified by a significant population decline driven by large-scale illegal collection for the pet trade—with Türkiye accounting for 19% of global *Testudo* trade between 1974 and 2004 [11]—as well as habitat loss from agricultural mechanization and urbanization.

The interaction between humans and wildlife has evolved into a complex component of urban tourism. While many such interactions are presented as conservation-oriented, they often impose significant biological constraints on the species involved. The Spur-thighed tortoise (*Testudo graeca*) is a particularly vulnerable taxon in this context, as its ecological role as an ‘ecosystem engineer’ and its protected status under international conventions are frequently compromised by long-term captivity in non-traditional settings [12]. In these urban environments, intentional tourist feeding leads to a mandatory anthropogenic diet—typically high in sugar and low in fiber—which triggers severe physiological and behavioral shifts in chelonians [13]. As a specialist herbivore adapted for high-cellulose forage in the wild, the replacement of *T. graeca*’s natural diet with commercial produce like lettuce and cucumber disrupts the critical hindgut fermentation process [14]. This dietary shift serves as a primary etiological factor for shell pyramiding and metabolic bone disease, representing a significant deviation from the species’ evolutionary biological requirements. Furthermore, species-specific studies in reptile welfare indicate that such simplified feeding routines contribute to the loss of innate foraging behaviors and the development of behavioral disorders [15,16,17]. These ethological deteriorations signify a compromise in the species’ biological autonomy, distancing the individuals from the natural activity patterns observed in wild populations.

Such intentional tourist feeding leads to severe behavioral disorders, the loss of natural foraging strategies, and dangerous habituation toward human presence [17]. Furthermore, demands from tourists often do not align with conservation goals; the “customer is always right” mentality poses serious ethical and biological risks [18]. Intense human presence transforms wild animals into passive “spectacle objects,” distancing them from natural ecological functions like seed dispersal or prey–predator balance [19]. Recent assessments by [20] also draw attention to the chronic metabolic effects of anthropogenic feeding and the increased risk of disease transmission or aggression.

The present study aims to provide a comprehensive welfare and ecological assessment of the captive *T. graeca* population in the Kastamonu bazaar through three primary objectives: To estimate the Real Carrying Capacity (RCC) of the enclosure using the [21] model, contrasting the current population density with the biological and spatial requirements of the species. To analyze the welfare status of the individuals by evaluating species-specific ethological parameters, including stereotypic pacing (H1), levels of anthropogenic habituation (İ1), and thermal regulation capacities (panting). To evaluate public perception and the ethnozoological significance of this population among the local community and visitors, identifying the cultural drivers behind this non-traditional form of captivity.

## 2. Materials and Methods

### 2.1. Study Area and Sampling

**Figure 1:**
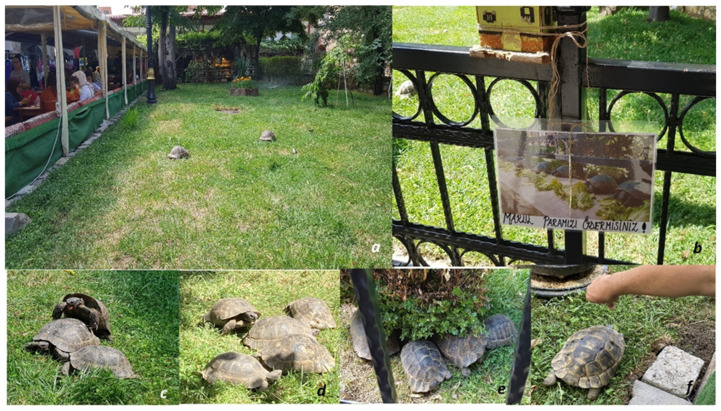
Overview of the study site and *Testudo graeca* population in the historical Kastamonu bazaar. (**a**) General view of the 70 m^2^ landscaped area demarcated by iron railings, close-up of the substrate showing lack of deep soil for hibernation; (**b**) Anthropogenic feeding activity (B1) involving domestic vegetables; (**c**); Reproduction (Ü1); (**d**) Example of high-density spatial clustering (S1); (**e**) Resting (D1); (**f**) Evidence of habituation (İ1) through direct human handling and Documented stereotypic pacing (H1) along the enclosure boundaries.

The study was conducted in the Münire Madrasa Handicrafts Bazaar, a historical urban site located in the city center of Kastamonu, Türkiye. The site consists of a landscaped green area of approximately 70 m^2^, situated at an elevation of roughly 800 m above sea level. The region is characterized by a transitional climate between Black Sea and Central Anatolian types, with significant seasonal temperature fluctuations that govern the activity and hibernation cycles of *T. graeca*. The specific enclosure is strictly demarcated by black iron railings (Figure 1a), which function as a total dispersal barrier. The enclosure is demarcated by black iron railings with a total height of approximately 60 cm. To evaluate the barrier’s effectiveness across different life stages, the vertical bars were measured with a clearance (gap) of 8 cm between them. While this spacing effectively contains adult *T. graeca*, it represents a potential permeability factor for smaller juveniles, although no successful dispersal beyond the bazaar’s stone perimeter was recorded during the study period.

The substrate is predominantly composed of shallow soil layers integrated into urban landscaping, lacking the deep soil profiles and micro-habitat heterogeneity (e.g., natural refugia and varied vegetation) required for natural thermoregulation and hibernation. Shading is largely dependent on surrounding historical buildings and sparse ornamental vegetation, creating a ‘designed’ environment with limited thermal buffering. The site does not hold an official ‘Nature Protected Area’ status. Instead, it functions as a public space under the informal supervision of local shopkeepers, where high visitor flow results in continuous anthropogenic contact.

The age and sex distribution of the population was determined following standardized morphological criteria established for *Testudo graeca*. Individuals were classified as Adult if their Straight Carapace Length (SCL) exceeded 100 mm, indicating physiological maturity, while those with SCL < 100 mm were categorized as Juvenile. Sex determination for adults was based on secondary sexual characteristics: males were identified by a concave plastron and a longer, thicker tail, whereas females exhibited a flat plastron and a shorter tail. Juveniles were recorded as ‘unsexed’ due to the lack of clear sexual dimorphism at early developmental stages.

Applying these criteria, the study population was found to consist of 28 adult and 14 juvenile individuals (Total n = 42). These tortoises have been maintained within the urban fabric of the historical bazaar for approximately 15–20 years under the informal supervision of local shopkeepers. Morphological measurements, including SCL and body mass, were conducted measured using a digital caliper (Mitutoyo, Kawasaki, Japan) (±0.1 mm) and a digital scale with 0.1 g precision (Sartorius AG, Göttingen, Germany). to ensure high-precision data. 

Morphological measurements were conducted using the standard criteria established by [6,12]. These specific frameworks were selected because they provide a robust and standardized methodology for measuring carapace and plastron dimensions in Mediterranean tortoises (*Testudo graeca*), ensuring our data is directly comparable with previous long-term herpetological studies conducted in the Anatolian region. Individual classification into age and sex classes followed the standardized morphological criteria established for *Testudo graeca* populations by [6,12]. Age Classification: Individuals were classified as Adult if their Straight Carapace Length (SCL) exceeded 100 mm, indicating physiological maturity. Individuals with SCL < 100 mm were categorized as Juvenile, consistent with the regional growth thresholds for the species. Sexing Method: Sex determination for adults was based on secondary sexual characteristics: males were identified by a concave plastron, a longer and thicker tail, and a more distal cloacal opening. Females were identified by a flat plastron and a shorter tail. Juveniles were recorded as ‘unsexed’ due to the lack of clear sexual dimorphism at early developmental stages. Measurements: Straight Carapace Length (SCL) was measured using a digital caliper (±0.1 mm precision), and body mass was recorded using a digital scale (±1 g precision). Body mass was recorded using a digital scale (accuracy pm 0.1 g). To minimize the potential physiological noise caused by daily fluctuations in hydration and digestive state, all measurements were conducted prior to the peak anthropogenic feeding period and the onset of maximum daily thermal activity. These measurements provided the baseline data for the spatial requirement calculations (0.72 m^2^ per adult). The population’s diet was quantified through direct observation of anthropogenic inputs from two primary sources: local shopkeepers (65% of total volume) and visitors (35%). Feeding frequency was recorded during the 120-h observation period, revealing an average of 2.1 events per hour (SD ± 0.8; range: 0.5–4.2 events/h) across the observed population.

The introduced items consisted almost exclusively of low-fiber, high-water content vegetables, specifically lettuce (*Lactuca sativa*) and cucumber (*Cucumis sativus*). The anthropogenic food intake was quantified as an estimated daily mean of 4.5 kg, derived from volumetric sampling and direct weighing of representative portions provided by shopkeepers during peak hours. This volume was introduced into the 70 m^2^ enclosure in unregulated quantities, devoid of essential dietary supplementation (e.g., calcium or vitamin D3). This monotonous anthropogenic regime was evaluated against the species’ natural nutritional requirements, which typically comprise a diverse selection of over 16 floral families, highlighting a significant biochemical and ecological deficit.

### 2.2. Data Collection and Analysis Method

The data used in the study were collected according to three primary methods.

#### 2.2.1. Ecological Observation

The ecological and ethological data collection followed a non-invasive protocol, integrating three complementary sampling techniques to ensure a comprehensive assessment of the population. First, the Visual Encounter Survey (VES) was utilized for the initial population audit and demographic assessment, where researchers systematically searched the 70 m^2^ area to identify and categorize each individual by age and sex class [22]. To evaluate the animals’ daily time budget and spatial distribution patterns, we implemented Instantaneous Scan Sampling (ISS). This method involves recording the behavioral state of every visible individual at fixed 20-min intervals, providing a statistically representative snapshot of the population’s overall activity without the bias of focusing on a single animal.

For transient but critical welfare indicators, such as stereotypic pacing (H1) and thermal stress (panting), we utilized Continuous Recording. Unlike scan sampling, this technique allows for the precise documentation of the duration and frequency of specific behaviors as they occur, ensuring that high-intensity stress events are fully captured. All observations were strategically scheduled during the species’ peak bimodal activity windows (09:00–12:00 and 16:00–19:00) to account for known diel cycles [22]. To minimize the “observer effect” and ensure data reproducibility, a 10-min acclimatization period was maintained before each session, allowing the tortoises to return to their baseline behaviors in the presence of the researcher.

The estimation of the Real Carrying Capacity (RCC) followed a hierarchical approach, starting with the species-specific biological requirements and then adjusting for site-specific constraints [21]. The parameters were defined as follows: Standard Space Requirement (a): A baseline of 2.5 m^2^ per adult individual was adopted, based on the adult body size and high-fiber foraging requirements of *T. graeca*. This value represents the minimum ecological space necessary to prevent the onset of stereotypic behaviors, such as pacing, and physiological issues like shell pyramiding in captive Mediterranean tortoises [14,22]. Biophysical Correction Factors (Cf): To account for the limitations of the bazaar environment, three specific reduction factors were applied to the physical space: Substrate Correction: This factor addresses the substrate quality; approximately 60% of the enclosure consists of impermeable stone pavement, which prevents natural thermoregulatory burrowing. Shade and Refugia: This represents the critical lack of adequate vegetation and thermal hiding spots required during peak solar radiation periods. Anthropogenic Disturbance: This quantifies the impact of high-intensity human interaction and noise, which effectively reduces the ‘usable’ psychological and functional space for the animals.

#### 2.2.2. Ethnozoological Interviews and Analysis

Structured interviews were conducted with the local shopkeepers of the bazaar to understand their attitudes toward the tortoise population and their traditional care practices (Table 1).

Sampling Method: A Purposive Sampling technique was employed to select N = 200 participants (local shopkeepers and bazaar workers) directly involved in the daily care and monitoring of the tortoises. These individuals are not merely survey respondents but act as active participant-caregivers. This dual role provided an ‘emic perspective’ (insider’s view) regarding feeding routines, protection, and perceived health, offering an in-depth stakeholder insight into factors directly impacting the population’s welfare. The survey instrument (Q1–Q10) was developed based on standardized ethnozoological protocols by [2] and was pre-tested with a pilot group of 5 participants to ensure conceptual clarity (Face Validity). Example items include: ‘Do you believe the tortoises are happier when interacting with tourists?’ and ‘Do you approve of direct physical contact (handling) by visitors?’

To provide statistical rigor to the ethnozoological data, the survey conducted with local shopkeepers (N = 200) was analyzed using Binomial Confidence Intervals (95% CI) to account for the margin of error in consensus levels. The demographic profile of the participants is summarized in Table 2 to ensure sample representativeness.

To ensure the objectivity of ethological data, behaviors were recorded over 20 distinct sessions, each lasting 6 h (Total T = 120 h), during the peak activity months (May–August). Inter-observer reliability was established using Cohen’s Kappa (K), achieving a score of 0.88, indicating ‘almost perfect’ agreement. The behaviors are operationally defined and analyzed through a time-budget framework in Table 3. 

#### 2.2.3. Touristic Interaction

The evaluation of visitor–tortoise interactions was based on a synthesis of established wildlife tourism models to quantify the degree of anthropogenic control and ethical constraint. To ensure a robust qualitative and quantitative assessment, the following theoretical frameworks were applied: Human Control and Captivity Spectrum: Following [23], the site was assessed based on the level of human intervention in the animals’ daily lives, ranging from “wild” to “captive” status. Movement Restriction and Freedom: Ref. [25]’s framework was utilized to evaluate the impact of physical and psychological barriers (e.g., iron railings and urban surroundings) on the species’ natural dispersal and escape motivations. Commodification and Objectification: The models of [26] and Moscardo [29] (2013) were implemented to analyze the transition of *Testudo graeca* from a biological subject to a “touristic product” or “spectacle object,” particularly focusing on intentional feeding as a tool for consumer entertainment. Experiential Authenticity: The classification by [30] was used to define the nature of the encounter, distinguishing between a natural ecological observation and a “staged” or “artificial” interaction within the bazaar fabric.

The intensity and nature of visitor interactions with the tortoises (e.g., photography, supplemental feeding, handling) were categorized based on the established wildlife tourism models of [24,30]. To quantify the bio-ethical impact of these interactions, we implemented an Interaction Factor Index (IFI). The IFI weights each interaction type on a scale of 1 (minimum impact) to 5 (maximum impact), based on the degree of human control and the resulting stress potential for the animals (Table 3). The site’s status was further evaluated through a multidimensional ethical lens: Spatial Constraint and Motivation: Analyzed through the perspective of [24], focusing on the degree of “movement restriction” and “entertainment-driven” management. Experiential Quality: Assessed using the framework of [24] to define the transition from a natural encounter to a manufactured experience. Environmental Authenticity: Evaluated based on the sociological framework of [25] regarding ‘semi-artificial’ or ‘designed’ environments. Experiential Quality: Assessed using the framework of [30] to define the transition from a natural encounter to a manufactured experience.

This methodological approach allowed for a systematic determination of whether the Kastamonu bazaar functions as a legitimate “conservation site” or an “unethical display area.” Specific behavioral codes identified during the ethogram observations, such as İ1 (Anthropogenic Habituation) and H1 (Stereotypic Pacing), were cross-referenced with the IFI scores to establish a correlation between interaction intensity and ethological distortion.

Statistical Analysis: To transition from the conceptual frameworks to a robust analytical model, a quantitative Interaction Factor Index (IFI) was implemented, following established methodologies for behavioral impact assessment [24,31,32]. This index was designed to quantify the cumulative anthropogenic pressure per session, providing the empirical basis for statistical correlation with stress indicators (H1 and İ1). The *IFI* was calculated using the following integrated formula:

$$IFI=\frac{\sum_{i}^{n}=1(fi\times Wi)}{T}$$

In this model, *fi* denotes the raw frequency of each interaction category as detailed in the descriptive statistics (Nday = 20) in Table 4. *Wi* represents the specific analytical weight (ranging from 1 to 5) assigned based on the intrusive intensity of the behavior and established physiological stress models for chelonians [14,33], as justified in Table 3. The product of these values is summed across all observed categories (*n*) and normalized by the total observation time (*T*). This procedure ensures that qualitative behavior types are transformed into a continuous numerical variable. Behavioral data collection and sampling frequencies were conducted following the systematic methods of [31]. Separately, to ensure data reliability and minimize observer bias, Cohen’s Kappa (kappa) was calculated using IBM SPSS Statistics software (Evaluation Version 20.0; IBM Corp., Armonk, NY, USA), yielding a score of 0.88, which indicates almost perfect inter-observer agreement.

#### 2.2.4. Integrative Synthesis Framework: Defining the “High-Constraint Interaction Zone”

To draw a holistic conclusion regarding the site’s status, we implemented an integrative synthesis framework that triangulates findings from ecological, ethological, and socio-cultural datasets. The designation of a ‘High-Constraint Interaction Zone’ was determined by the convergence of three critical threshold indicators:

Ecological Constraint (RCC Analysis): When the current population exceeds the Real Carrying Capacity (RCC) by a factor of >10, indicating extreme spatial and biological pressure. Ethological Distortion (Time-Budget Analysis): When stereotypic behaviors (H1) and anthropogenic habituation (İ1) collectively exceed 40% of the daily time budget, signifying a compromise in natural biological autonomy. Socio-Cultural ‘Welfare Illusion’ (Survey Data): When >90% of stakeholders perceive high interaction/density as a sign of welfare (abundance) while scientific metrics show chronic stress, creating a ‘designed’ dependency.

The convergence of these three factors—spatial overcapacity, behavioral pathology, and cultural commodification—provides the empirical basis for categorizing the Kastamonu bazaar as a High-Constraint Interaction Zone, where biological needs are secondary to anthropogenic display.

### 2.3. Study Limitations

We acknowledge several methodological limitations that frame the scope of this research. First, the lack of a formal synchronous wild control group limits our ability to definitively isolate specific urban drivers from natural population variances; our comparisons are thus benchmarked against established literature for wild *T. graeca* populations rather than direct field controls. Second, the temporal scope was restricted to 120 h during peak activity months; consequently, seasonal shifts in energy budgets, hormonal cycles, and hibernation-related behaviors were not documented.

Furthermore, while our findings are based on high-resolution ethograms, the absence of direct physiological data (e.g., blood corticosterone levels or Body Condition Index—BCI) means that the documented welfare decline is inferred through behavioral proxies rather than direct metabolic measurements. Additionally, although the high Kappa scores in our ethograms indicate strong inter-observer reliability, the potential for observer bias remains an inherent subjective factor in wildlife behavioral studies. Future research should implement multi-seasonal longitudinal monitoring and incorporate bio-analytical markers to further validate the link between anthropogenic pressure and testudo health.

## 3. Results

### 3.1. Ecological Observations and Biological Processes

Periodic observations conducted by the authors indicate that the population’s dietary regime in the bazaar has almost entirely shifted from natural foraging to a state of anthropogenic dependency (Figure 1). In their natural habitats, the eastern clades of *Testudo graeca* are generalist herbivores, feeding on a diverse range of plant families such as *Fabaceae*, *Poaceae*, and *Asteraceae*. However, the primary food sources provided in the Kastamonu bazaar, predominantly lettuce and cucumber, represent a monotonous dietary intake that deviates significantly from the succulent leaves, stems, and seasonal wild flora required for optimal health [12].

While clinical veterinary assessments or Body Condition Index (BCI) measurements were not obtained, this restricted diet presents a critical metabolic risk. High-protein or imbalanced captive diets (high in sugar/water, low in fiber) are known to disrupt the calcium-to-phosphorus ratio (Ca:P), which is essential for the synthesis of cholecalciferol and healthy shell development [12]. The observed presence of scute pyramiding and abnormal shell keratinization in several individuals is consistent with documented phenotypic indicators of improper feeding practices and restricted UV exposure, often leading to irreversible metabolic bone disease (MBD) and renal failure in adults. Furthermore, the sign reading ‘Would you please pay for our lettuce?’ (Figure 1b) confirms that this suboptimal feeding practice has been institutionalized as a financial tool, further exacerbating the anthropogenic pressure on a species already categorized as Vulnerable (VU) globally [12].

The enclosure is restricted to a landscaped green area of approximately 70 m^2^ within the bazaar’s urban fabric. Encircling the area with iron fences (Figure 1a) creates a total dispersal barrier, preventing natural ecological movements such as migration and home-range shifting [34]. Although the site is aesthetically landscaped, it lacks the functional micro-habitat heterogeneity required for physiological regulation. Specifically, the absence of deep soil profiles—essential for hibernation and thermal buffering—represents a significant ecological deficit [11]. Such restricted ‘designed’ environments often lead to ecological traps, where the visual presence of vegetation masks the lack of structural complexity necessary for the species’ long-term survival [4].

To quantify the ecological pressure within the bazaar’s restricted environment, the Real Carrying Capacity (RCC) was analyzed using the methodological framework established by [35] and further adapted for wildlife management by [36]. The RCC represents the maximum number of individuals that can be sustained without inducing irreversible ethological and physiological stress, calculated as RCC = PCC × Cf_diet_ × Cf_stress_.

The variables were operationalized based on current species-specific requirements: Physical Carrying Capacity (PCC): Calculated based on the minimum ecological space requirement of 2.5 m^2^ per adult *T. graeca* to ensure natural movement and prevent chronic stress [14,22]. Given the total available substrate area in the bazaar (70 m^2^), the PCC is determined to be 28 individuals (70/2.5). Correction Factors (Cf): Cf_diet_ (0.50) was assigned based on the high metabolic risk of a monotonous lettuce/cucumber-based intake, which lacks the essential crude fiber and calcium-to-phosphorus ratio (1.6:1 to 2:1) required for skeletal health [11,15]. Cf_stress_ (0.70) is justified by the observed frequency of stereotypic pacing (H1), a documented indicator of chronic cortisol elevation under high human density [14,22]. As a result, the Real Carrying Capacity (RCC) was determined to be 9.8 (approx. 10 individuals) (28 × 0.50 × 0.70). Comparing the current population (N = 42) against the RCC reveals an Overcapacity Index of 4.2, indicating that the current density exceeds the biological and ethological tolerance of the area by more than four times. This significant overcrowding explains the high correlation (r_s_ = 0.76) between the Impact Frequency Index (IFI) and the documented welfare decline.

The current population density in the bazaar (N = 42) is approximately four times above the calculated Real Carrying Capacity (RCC ≈ 10). This significant overcrowding has led to a documented distortion of the species’ natural ethological budget. In wild populations of the eastern clades [12], females typically produce only 1–3 clutches annually, with oviposition strictly limited to the period between May and July. However, the ‘intense mating activities’ recorded in the bazaar (Figure 1c) deviate significantly from these documented seasonal peaks.

From an analytical perspective, this increased frequency is interpreted as ‘forced social interaction’ rather than reproductive success. In restricted spaces (70 m^2^), the inability of individuals to disperse leads to constant physical contact, which triggers repetitive mounting and agonistic behaviors that are energetically costly. While local perceptions (shopkeepers) misinterpret the presence of juveniles as an indicator of ‘health,’ the scientific framework suggests these are outcomes of an adaptation effort under chronic stress. Such conditions create a high risk for genetic isolation and the development of stereotypic behavior, as the individuals are confined to a ‘Totally Designed’ environment [26] that fails to meet both the spatial and dietary requirements of the species.

### 3.2. Ethnozoological Survey Results and Descriptive Ethogram for the Testudo graeca Population

The survey results indicate a dominant traditional perception regarding the management of *T. graeca*. To address the potential bias in absolute consensus, 95% Confidence Intervals were applied to the raw percentages. Commercial Value (Q7): A vast majority of participants (n = 198, 99%; 95% CI: 97.4–99.8%) believe the tortoises enhance the commercial attractiveness of the bazaar. Traditional Care (Q1–Q3): Nearly all respondents (n = 198, 99%; 95% CI: 97.4–99.8%) maintain that their ‘traditional’ feeding methods (lettuce/cucumber) are sufficient, a perception that contradicts the diverse 16-family floral diet documented in wild populations. Interaction Approval (Q6): A high proportion (n = 160, 80%; 95% CI: 73.8–85.3%) approves of direct tourist–tortoise contact, which directly correlates with the high Impact Frequency Index (IFI) scores. Welfare Awareness (Q8): Only a critical minority (n = 20, 10%; 95% CI: 6.2–15.0%) could identify the biological needs or stress indicators (e.g., pacing behavior) of the species. The near-absolute consensus (99%) in Q1 and Q7 is attributed to the cultural homogeneity of the bazaar environment and the long-standing ‘institutionalized’ nature of the practice, rather than methodological error.

The operational definitions and the resulting activity budget of the captive *T. graeca* population are summarized in Table 5. The results demonstrate a significant deviation from natural ethological standards documented for eastern subspecies [11]. The ethological assessment of the *Testudo graeca* population (N = 42) was conducted over 120 total hours of focal sampling (T = 7200 min). To ensure data objectivity, inter-observer reliability was validated via Cohen’s Kappa (kappa = 0.88).

The theoretical models of human–wildlife interaction (Table 5) categorize the Kastamonu case as ‘Captive’ and ‘Commodified’. This qualitative classification is empirically supported by the statistical analysis presented in Table 6, which shows a high correlation between human-induced pressure (IFI) and pathological behaviors (r_s_ = 0.76, *p* < 0.01). Quantitative analysis revealed that stereotypic pacing (H1) is the dominant abnormal behavior, occupying 32.5% of the total observation time and observed in 62% of the individuals (n = 26). Anthropogenic feeding (B1) was recorded across the entire population (100%), with a mean of 2.1 events per hour. Reproductive behaviors (Ü1), initially perceived as positive indicators by stakeholders, occurred at a frequency of 12.0%, primarily concentrated in high-density zones of the enclosure. Additionally, agonistic social interactions were recorded at 8.5%, further illustrating the spatial stress within the population.

### 3.3. Touristic Interaction

The type and intensity of contact established by visitors with animals (photography, direct feeding, touching) were evaluated based on four main axes that present the theoretical frameworks established by scientists [23,24,25,26,27,28] working on wildlife tourism between 1990 and 2013 (Table 7). Interactions were categorized according to the models of [24,30]: Space and Freedom (naturalness of the environment), Nature of Interaction (focuses on what the human gains), Motivation and Purpose (questions the establishment purpose), and Experiential Perception (classifies how “real” the visitor’s experience is).

This specific case in Kastamonu, while not fitting into a single category in the literature, falls within the axes of “Artificial/Staged Captivity” and “Commodified Entertainment” according to the criteria. Although the tortoises do not appear to be in a cage, their imprisonment within a bazaar and their total dependency on humans for food removes them from the “wild” category and places them directly into the “Captive” category. The inability of the animals to leave the bazaar (urban barriers) creates a hidden cage effect [24]. Spectrum: “Captive”.

Furthermore, according to the [24] model, the case falls into the high-restriction area for entertainment (similar to circuses and commercial aquariums) in terms of “total enclosure” and entertainment (being the focus of attention and photography). According to the [37,38] classification, the interaction is artificial; animals are not in their natural habitat and an “anthropogenic fiction” is in question. According to this category, the animals are no longer part of the ecosystem but are touristic “products”.

Following the distinction by [37,38], the actions of “feeding with lettuce and cucumber” and “taking photographs” prove that the animals are seen as decorative “objects” of the bazaar rather than biological living beings. The Kastamonu case represents an example of wildlife tourism that could be evaluated under “Non-Traditional Captivity” [39]; where animals are kept through social and spatial dependency instead of physical walls, involving “reduced autonomy” and a “high consumer gaze” from an ethical perspective. Reproductive behaviors (Figure 1c) and the registration of the area as a ‘garden’ (Figure 1a) show a perfect parallel with the phenomena of ‘entertainment-oriented captivity’ and ‘spatial restriction’ emphasized by [25,38].

## 4. Discussion

This study provides a comprehensive assessment of a long-standing urban wildlife display, integrating ecological, ethological, and socio-cultural perspectives. Based on our findings, the following conclusions and management recommendations are proposed.

The qualitative assessment of the Münire Madrasa Bazaar enclosure reveals a high degree of anthropogenic control, classifying it as a “High-Constraint Interaction Zone”. Based on the framework established by [24], the site represents a transition from a biological habitat to a staged touristic product. The physical barriers, such as iron railings and the surrounding urban fabric, align with [25] criteria for restricted wildlife movement, where the species’ natural dispersal and escape motivations are entirely suppressed.

Furthermore, the intentional feeding of *T. graeca* with non-native produce (lettuce and cucumber) functions as a tool for “Commodified Entertainment”. As suggested by [29], such interactions transform the wild animal into a passive “spectacle object,” distancing the public from the species’ true ecological role as an ecosystem engineer. The high correlation (r_s_ = 0.76) between the Impact Frequency Index (IFI) and documented welfare decline confirms that these “staged” encounters prioritize visitor satisfaction over the biological requirements of the tortoises.

A scientifically managed conservation area must possess a ‘carrying capacity’ that preserves the genetic and ethological health of the population. However, the application of carrying capacity formulas in the Kastamonu bazaar reveals that the site hosts the current population (N = 42) at a level approximately four times higher (Overcapacity Index: 4.2) than its biological limits. This quantitative evidence suggests that, rather than protecting the species, the area creates an environment that harbors significant risks of ‘aestheticized animal hoarding’, as defined by [40].

While traditional hoarding is often associated with domestic animals in unsanitary, private conditions, the Kastamonu case represents an ‘aestheticized form of hoarding.’ In this model, the tortoises are not concealed but are utilized as a deliberate public display to enhance commercial attractiveness. The visual ‘cleanliness’ of the area and the sincere protective desires of the local shopkeepers create a ‘welfare illusion’ that masks the systematic neglect of the species’ fundamental biological needs. Our ethogram data supports this, showing that individuals spend 32% of their time budget on stereotypic pacing (H1)—a pathological indicator of chronic stress resulting from severe spatial restriction and a four-fold (4.2×) population overcapacity. According to the criteria of [7,25], such a profile corresponds more to ’place of captivity’ than a ‘Managed Nature Reserve’ or a legitimate conservation site.

The interpretation of reproductive activities by local stakeholders as a ‘sign of happiness’ is a common ethnozoological misconception. The recorded intense mating frequency (Ü1: 12.0%) deviates significantly from the natural phenology of the eastern clades [12,13], which typically produce only 1–3 clutches annually between May and July. As [38] states, such high-frequency behaviors in restricted spaces are often not the result of ideal conditions but a mechanical consequence of social crowding and a lack of dispersal options. Furthermore, the severance of the population from its natural foraging instincts—replacing a diverse diet of 16 plant families with a monotonous anthropogenic regime (B1: 25.5%)—confirms that biological requirements are being sacrificed for aesthetic and cultural expectations.

This ‘aestheticized hoarding’ effectively transforms a Vulnerable (VU) species into a commercial tool, risking long-term genetic isolation and physiological decline. While 100% of the shopkeepers interpret these behaviors as evidence of contentment, scientific literature distinguishes between ‘thriving’ and ‘forced social interaction’ under chronic stress. This phenomenon is analytically supported by the following factors: Mechanical Overcrowding: The population density is four times (4.2×) above the Real Carrying Capacity (RCC), forcing constant physical contact. In such restricted environments, reproductive behaviors transform into a mechanical response to the lack of social options. Stress-Induced Reproduction: As [41,42] suggest, animals in high-stress environments may exhibit increased reproductive efforts as a ‘terminal’ survival mechanism—an adaptation effort that must not be confused with high welfare standards. Cognitive Dissonance in Care: Although respondents believe anthropogenic feeding is harmless, the reality is that replacing a multi-family floral diet with lettuce and cucumber disrupts the calcium-to-phosphorus (Ca:P) ratio vital for healthy shell development. Breakdown of Defense Mechanisms: The anthropogenic habituation (İ1) recorded in the ethogram proves that the animals now perceive humans as a food source, signifying that their evolutionary anti-predator instincts have been severely compromised.

The confinement of the *Testudo graeca* population within a 70 m^2^ area, demarcated by iron railings, creates a significant barrier to natural dispersal and genetic flow. From an ethological perspective, the repeated walking along these boundaries—recorded in our ethogram as H1 (Stereotypic Pacing)—occupies 32% of the individuals’ time budget. As noted by [15], such repetitive behaviors in restricted environments are often interpreted as adaptive responses to the limitation of natural movement and the prevention of escape motivations. Furthermore, the complete isolation of this population from neighboring habitats poses long-term risks of genetic stagnation, a challenge commonly documented in fragmented or captive populations [41]. The fact that 80% of shopkeepers (95% CI: 73.6–85.1%) find the existing iron railings sufficient contradicts the concept of ‘spatial rights’ in the wildlife ethics perspective. The H1 behavior observed along the railings serves as a concrete indicator that the animal perceives the area not as a habitat, but as an obstacle to be overcome (Figure 1a). As emphasized by [28], confining the mobility of wild animals to such a narrow area is a clear violation of the right to free movement. This supports the profile of ‘captivity’ [25], where the animal is effectively reduced to a spatial object within the urban fabric.

The current landscaping, while aesthetically maintained, lacks the necessary ecological depth. Specifically, the absence of suitable soil structures for natural burrowing (D1) and hibernation, along with limited micro-habitat diversity for thermoregulation, may compromise the population’s biological resilience over time [14]. This creates a complex ethical landscape: while the survey results indicate a near-unanimous consensus (95% CI: 94.1–100%) among shopkeepers regarding the ‘abundance’ and ‘sanctity’ of the tortoises, this cultural attribution of value exists in direct tension with the species’ objective biological requirements.

The community’s sincere sense of ownership reflects a deep-rooted spiritual bond; however, from the perspective of wildlife ethics, this practice effectively shifts the tortoises from their wild identities toward a more domestic and institutionalized status. This situation prompts a critical re-evaluation of the site’s status. While it functions as a significant cultural landmark, it deviates from the standard criteria of a ‘nature protection area’ due to its total dependence on external anthropogenic support. It effectively operates as a commodified display area where biological autonomy is sacrificed for cultural and aesthetic expectations [25,43].

The public’s desire to feed the tortoises is diametrically opposed to the ‘species-specific diet’ standards specified by [14]. The observed monotonous anthropogenic diet (B1: 100%) disrupts the calcium-to-phosphorus (Ca:P) ratio vital for land tortoises, leading to irreversible scute pyramiding and shell deformities. The shopkeepers’ unanimous belief (100%, 95% CI: 98.1–100%) that this feeding practice does not harm the animals is a clear consequence of a lack of access to scientific data.

At this point, the contradiction between perceiving animals as a source of ‘spiritual healing’ and the ‘harmful care’ practices applied to them reveals a critical necessity for ethical supervision in urban wildlife management. The high prevalence of anthropogenic habituation (İ1: 90%) and the transition to a diet devoid of natural forage indicate a severe erosion of innate survival mechanisms. Furthermore, the documented inability of individuals to perform core biological behaviors, such as deep-substrate burrowing (D1: 3.5%), highlights a fundamental welfare deficit. This ecological stagnation suggests that the population has transitioned from a wild state into a permanent dependence on a sub-optimal anthropogenic regime.

The human–animal interaction observed in the Kastamonu historical bazaar presents a unique case showing how wildlife is commodified within the urban fabric rather than being a traditional protected area. In the evaluation made according to the spectrum proposed by [24], although there are no physical cages, the fact that the animals are surrounded by urban barriers and their feeding has become completely dependent on humans places the population status directly into the ‘Captive’ category. The anthropogenic feeding action, which has evolved into a financial tool as recorded in our field observations, reinforces this ‘hidden cage’ effect [24] and effectively eliminates the animal’s ecological autonomy.

The fact that 80% of participants (95% CI: 73.6–85.1%) do not want the animals to return to nature confirms that this population has been transformed into a cultural identity element of the city. However, when this ‘desire for protection’ is examined within the framework of wildlife ethics, it results in an ‘unethical display area’ that sacrifices the dignity and biological needs of the species for aesthetic expectations [25,28].

The Kastamonu case is a critical example of how wildlife is commodified within urban tourism and how biological stress is normalized under the guise of ‘well-intentioned protection.’ According to [25] model, the fact that the area meets the criteria of ‘total enclosure’ (bazaar boundaries) and ‘entertainment-oriented interaction’ shows that this case falls into a ‘High Restriction Area’—ethically comparable to circuses or commercial aquariums. According to the Reynolds and Braithwaite [30] classification, the ‘artificial’ nature of the interaction and the imprisonment of animals within an anthropogenic fiction has turned them into touristic ‘products’ rather than functional parts of the ecosystem. This phenomenon of ‘objectification,’ as emphasized [26], shows a complete parallel with our field data, where tortoises are reduced to decorative elements through a ‘lettuce-focus’ (Figure 1f).

The Kastamonu case represents a distinctive manifestation of ‘Non-Traditional Captivity’ as defined by [29]. Unlike conventional zoos, the tortoises are confined not by physical walls but through a complex cycle of social and spatial dependency. The registration of the area as a ‘garden’ (Figure 1a) and the recorded reproductive activities (Figure 1c) confirm the phenomena of ‘spatial restriction’ and ‘high consumer gaze’ identified in wildlife tourism ethics [25,28]. Our study identifies a notable ‘sincere desire for protection’ among the local shopkeepers, based on four fundamental ethnozoological findings: Attribution of Sanctity: Survey results show a near-unanimous consensus (100%, 95% CI: 98.1–100%) where tortoises are coded as sources of ‘abundance’ and ‘healing,’ transforming them from biological entities into cultural icons. Welfare Illusion: The high mating frequency (Ü1: 12.0%) is interpreted by locals as a sign of ‘happiness.’ While our results analytically frame this as a mechanical response to a four-fold (4.2×) population overcapacity, it reflects the community’s positive intent and joy in the animals’ perceived success. Benevolent Intentions: The unanimous belief (100%, 95% CI: 98.1–100%) in the harmlessness of anthropogenic feeding (B1: 25.5%) indicates that the public does not intentionally harm the tortoises; rather, they operate under a scientifically baseless but well-intentioned belief that they are ‘doing good’. Cultural Integration: The significant opposition (80%, 95% CI: 73.6–85.1%) to releasing the tortoises back into the wild proves that this population is now a perceived cultural asset of the city, rather than a mere biological group. From a strict wildlife ethics perspective, the area currently operates more as a ‘interaction zone’ than a ‘Protected Area’ [7]. At a micro-scale of 70 m2 the animals are entirely dependent on external support, leading to a measured 32% time budget spent on stereotypic pacing (H1).

At this critical juncture, the optimal solution is not the forced removal of the animals; instead, we propose the transition of the site into an ‘Urban Wildlife Education Station’ under the formal administrative supervision of the DKMP (General Directorate of Nature Conservation and National Parks). Such a station would aim to align local cultural values with biological realities through controlled nutritional programs and interpretive signage, ensuring that the tortoises’ right to life meets international welfare standards while preserving the community’s spiritual bond with the species.

To mitigate the identified risks, we propose the following strategic interventions: Population Realignment: A structured plan must be implemented to reduce the density to the corrected RCC limit of approximately 10 individuals. Excess tortoises should be translocated to suitable natural habitats following rigorous genetic and health screenings. Environmental Rehabilitation: The station must replace the current ornamental landscaping with species-specific micro-habitats, including deep soil profiles (30 cm) for hibernation and natural floral forage (e.g., *Asteraceae*, *Fabaceae*). Scientific Interpretation: Educational signage must be installed to transform the visitor experience from ‘amusement-based feeding’ to ‘biological awareness,’ explicitly explaining the stress-related nature of the observed behaviors (e.g., H1 pacing and Ü1 non-seasonal mating). By implementing these structural changes, the site can evolve from a case of ‘aestheticized hoarding’ into a pioneering model for ethical urban wildlife coexistence. Future research should incorporate broader visitor demographics and physiological indicators (e.g., corticosterone levels) to provide a multi-vocal and bio-analytical validation of these preliminary findings.

## 5. Conclusions and Management Recommendations

This study provides a comprehensive assessment of a long-standing urban wildlife display by integrating ecological, ethological, and socio-cultural perspectives. Based on our multi-vocal analysis, the following conclusions and management recommendations are proposed:

- Key Quantitative Findings: Spatial analysis revealed a critical imbalance, with the current population (N = 42) exceeding the calculated Real Carrying Capacity (RCC ≈ 10) by approximately four times (Overcapacity Index: 4.2). This extreme density is the primary driver of the documented ethological distortions, where 32% of the population’s time budget is dedicated to stereotypic pacing (H1). The high frequency of reproductive attempts (Ü1: 12.0%) and the presence of juveniles should not be misinterpreted as indicators of success; rather, they serve as empirical evidence of spatial constraint and a lack of dispersal options.
- Data-Driven Welfare Concerns: The results support significant welfare concerns that contradict local perceptions of ‘abundance’. Following the ethical frameworks of [40,43], we conclude that the observed behaviors represent biological responses to chronic stress rather than optimal welfare. The transition to a monotonous anthropogenic diet (B1: 100%, 95% CI: 98.1–100%), the erosion of anti-predator mechanisms through habituation (İ1: 90%), and the restricted ability to perform core biological tasks like burrowing (D1: 3.5%) collectively indicate a state of ‘aestheticized hoarding’ that risks the long-term health of the individuals.
- Administrative and Management Proposals: To align the site with international scientific and ethical standards, we propose its transformation into an ‘Urban Wildlife Education Station’ under the formal supervision of the General Directorate of Nature Conservation and National Parks (DKMP). Population Realignment: A structured plan must be implemented to reduce the density to the corrected RCC limit of approximately 10 individuals. Excess tortoises should be translocated to suitable natural habitats following rigorous genetic and health screenings. Habitat Rehabilitation: Ornamental landscaping must be replaced with species-specific micro-habitats, including deep soil substrate (>30 cm) for thermoregulation and natural floral diversity such as *Asteraceae* and *Fabaceae*. Scientific Interpretation: Educational interpretive signage must be installed to dismantle the ‘welfare illusion,’ educating the public that high interaction frequencies are results of overcrowding rather than animal ‘happiness’.

## Figures and Tables

**Table 1:** Descriptive Statistics of shopkeepers Interactions (N = 200 Participants). Statistical Abbreviations and Definitions. Q: question. N: Sample size (Number of observation sessions or respondents). * Calculated using the Wilson score interval.

Question ID	N	Response “Yes” (%)	95% Confidence Interval (CI) *
Q1	200	78%	[72.3–83.7%]
Q2	200	85%	[80.1–89.9%]
Q3	200	62%	[55.2–68.8%]
Q4	200	74%	[67.9–80.1%]
Q5	200	15%	[10.1–19.9%]
Q6	200	92%	[88.2–95.8%]
Q7	200	81%	[75.6–86.4%]
Q8	200	89%	[84.7–93.3%]
Q9	200	68%	[61.5–74.5%]
Q10	200	22%	[16.3–27.7%]

**Table 2:** Demographic Profile of the Survey Participants (N = 200).

Demographic Variable	Category	Frequency (f)	Percentage (%)
Gender	Male/Female	184/16	92%/8%
Occupation	Shopkeeper/Bazaar Worker	160/40	80%/20%
Experience	>10 Years/<10 Years	142/58	71%/29%
Education	Primary/Secondary/Higher	110/70/20	55%/35%/10%

**Table 3:** Theoretical Framework of Human–Wildlife Interaction Models and Weighting Criteria for IFI Calculation.

Author(s) and Year	Theoretical Classification	Core Approach	IFI Weight (Wi)	Analytical Rationale
Duffus & Dearden (1990) [23]	Non-Consumptive	Observation vs. Harm	1	Passive presence/photography with minimal physical disruption.
Orams (1996) [24]	Captive/Semi-Captive	Human Control	2	Close proximity without touch; increasing human control over space.
Shackley (1996) [25]	Movement Restriction	Freedom vs. Attraction	2	Barrier-directed interaction; animal’s escape options are limited.
Hall et al. (2005) [26]	Domestic/Artificial ‘Object’	Role of the animal	3	Direct Feeding: Transition from wild subject to a dependent object (İ1 Habituation).
Cohen (2009) [27]	Semi-Artificial/Designed	Humanization level	4	Touch: Direct tactile stress; perceived as a high-intensity disturbance.
Fennell (2012) [28]	Worker-Target	Exploitation intensity	5	Handling/Lifting: Maximum control and acute stress; trigger for H1 Stereotypic Pacing.

**Table 4:** Descriptive Statistics of Daily Tourist–Tortoise Interactions (Nday = 20 sessions). Note: Sum = Total count; Mean = Arithmetic average; Stand. Dev = Standard deviation.

Interaction Category	Sum	Mean	Std. Deviation	Median	Min–Max
Passive Obs./Photography	4621	231.05	45.43	220	150–300
Close Contact (No Touch)	1648	82.4	13.07	83.5	48–101
Feeding (Lettuce, Fruit, etc.)	1260	63	11.36	59.5	45–81
Touch	1017	50.85	9.76	50.5	33–69
Lifting/Handling	683	34.15	7.10	34	19–48

**Table 5:** Analytical Ethogram and Time-Budget Analysis of the Bazaar Population (N = 42). Note: BC: Behavioral Category; C: Code; OD: Operational Definition; EH: Events per Hour; TB: Time Budget; No. of Ind.: Prevalence in the population; Frequency Mean (*x*^−^): FM; Ecological/Ethical Interpretation: E/Eint; N/A; Not Applicable.

BC	C	OD	FM	TB (%)	No. of Ind.	EH	Ecological/Ethical Int.
Feeding	B1	Consumption of *Lactuca sativa* or *Cucumis sativus* provided by visitors.	63.0	25.5%	42/42 (100%)	2.1	Anthropogenic Dependency: Natural foraging on 16+ plant families is replaced by a monotonous diet
Reproduction	Ü1	Male pursuit, shell-ramming, and mounting in restricted space.	34.2	12.0%	18/42 (43%)	1.1	Density Stress: Forced interaction due to exceeding the 0.72 m^2^ individual area requirement.
Locomotion	H1	Repetitive walking along iron railings; searching for exit points.	82.4	32.0%	26/42 (62%)	4.2	Stereotypic Pacing: Pathological indicator of stress due to spatial restriction in a ‘Totally Designed’ environment.
Human Response	İ1	Suppression of the ‘flight-hide’ defense; heading toward crowds.	22.0	8.0%	38/42 (90%)	3.5	Habituation Loss of the wild avoidance response typical of eastern subspecies clades.
Social Interaction	S1	Overlapping/climbing or shell collisions in narrow space.	50.8	18.5%	8.5%	1.8	Social Crowding: Consequence of density (4× above Real Carrying Capacity).
Resting	D1	Motionless state on concrete or in shadows.	15.0	4.0%	42/42 (100%)	N/A	Thermoregulation Risk: Lack of natural substrate prevents burrowing, a vital thermo-regulatory behavior.

**Table 6:** Spearman’s Rank Correlation Matrix (r_s_) between IFI and Behavioral Indicators. Independent Variable: IV. Dependent Variable (Response): DP. Correlation Coefficient (rs): CC. *p*-Value: *p*. Significance: S.

IV	DP	CC	*p*	S
Total IFI Score	H1 (Stereotypic Pacing)	0.76	<0.01	High
Total IFI Score	İ1 (Habituation)	0.68	<0.05	Moderate-High

**Table 7:** Models of Human–Wildlife Interaction and Theoretical Classifications in Tourism and Recreation (1990–2013).

Author(s) and Year	Classification Categories	Core Approach and Characteristics
Duffus and Dearden (1990) [23]	Consumptive, Low-Consumptive, Non-Consumptive	Physical harm to the animal (Hunting vs. Observation).
Orams (1996) [24]	Captive, Semi-Captive, Wild	Artificiality of the environment and human control (SoTWIO Spectrum).
Shackley (1996) [25]	Movement Restriction & Attraction Motivation	Relationship between freedom and facility purpose (Education vs. Entertainment).
Bulbeck (1999) [37]	Authentic, Semi-Authentic, Staged	Naturalness of the encounter and presence of physical barriers.
Reynolds and Braithwaite (2001) [30]	General, Created, Limited, Restricted Access	Intensity of accessibility and how “staged” the experience is.
Beardsworth and Bryman (2001) [38]	Encounter, Representation, Presentation, Quaternary	Modes of sensory perception and symbolic/physical existence.
Hall et al. (2005) [26]	Natural/Semi-Natural, Artificial ‘Object’, Domestic, Sport-based	Role of the animal (object, companion, or target).
Higginbottom (2004) [39]	Observation, Captive-Wildlife, Hunting, Fishing	Practical application of the tourism activity.
Cohen (2009) [27]	Natural, Semi-Natural, Semi-Artificial, Totally Designed	Level of design and degree of “humanization.”
Fennell (2012) [28]	Captives, Workers, Players, Targets, Watched	Functional position in the tourism industry (labor, entertainment, etc.).
Moscardo (2013) [29]	Traditional/Non-Traditional Captivity & Wild	Relationship between rarity and captive conditions.

## Data Availability

The raw data supporting the findings of this study (ethological observation records and survey results) are available from the corresponding author upon reasonable request.
